# Book Review

**Published:** 1933

**Authors:** 


### Reviews of Books

**The Early History of the Infant Welfare Movement.** By G. F. McCleary, M.D., D.P.H. Pp. xii., 176. London: H. K. Lewis & Co. Ltd. Price 6s.—One of the direst results of the Industrial Revolution in England was an increasing indifference to the value of infant life. Some people look back with pride to the improvement which nineteenth-century legislation brought in the care of children; but many more must look back with contempt upon the conditions which rendered legislation necessary to restrict what we can only call the child destruction organized by the industrialists. For it is clear from Dr. McCleary's *Early History of the Infant Welfare Movement* that the individual exploiters of child labour were responsible rather than the system under which they made their fortunes. With the employers disregarding the welfare and health of children, it was not surprising that they bred a similar indifference in the parents, whom they equally exploited. Even the Prevention of Cruelty to Children Act only dates from 1889, and it is still impossible to prevent milk-vendors from distributing bovine tuberculosis where they please. To write temperately after reading Dr. McCleary's book is impossible. Although, as he shows, great progress has been made, there is little enough reason to be vain of our past achievements. In Great Britain we have just started looking after our babies: how difficult progress has been and against what difficulties the pioneers in every country have had to contend is made evident in this book. Nothing reflects greater discredit upon us as a nation than the discovery, from Dr. McCleary's book, that most of the medical leaders of the Infant Welfare Movement in England have received no public recognition of their labours. Unless, perhaps, the removal of Edwin Chadwick from the General Board of Health was a more striking tribute to his life work than his removers intended. The book is one of absorbing interest, which its author has made most attractively readable.

**A Shorter Surgery.** Third Edition. By R. J. McNeill Love, M.S. Pp. viii., 413. 96 illustrations. London: H. K. Lewis & Co. Ltd. 1932. Price 16s.—The medical student of the present day is expected to absorb and retain a vast amount of theoretical knowledge, often at the expense of his practical work. Any book which will help to curtail the total time expended in reading, and so allow a little time for the clinical study and examination of cases, must be a welcome addition to medical literature for students. *A Shorter Surgery* supplies the need for a book of the type indicated, it is indeed so short that in parts the text consists of little more than a list of facts. The author has no time or space for the discussion of controversial matters, consequently it is essential that this small volume be read in conjunction with a more complete work, as stated in the preface. There are one or two serious omissions. The classifications are good, and should be of the greatest help to the reader in framing lucid answers to examination questions, and should also train him to arrange what he wishes to write in a concise though readable form. The illustrations are excellent, and take the form of line drawings and photographs, both of cases and of X-rays. Short books on long subjects must always be read with caution, but the fact that this is now in its third edition should dispel any doubts as to its usefulness, and is ample evidence that the author has efficiently accomplished a difficult task.

**Common Skin Diseases.** By A. C. ROXBURGH, M.D. Pp. xxx., 322. Illustrated. London: H. K. Lewis & Co. Ltd. 1932. Price 18s.—Dr. Roxburgh is to be congratulated on the production of this text-book, which should be of value to students and general practitioners. Although a large number of books on dermatology have already been published, this one has certain features which will especially commend it. The index of preliminary diagnosis, together with the chapter on signs, symptoms and general diagnosis, should enable students to determine the group to which any particular case belongs. Details of treatment which can be carried out in general practice are given, and the indications for those forms of treatment which require special apparatus and experience, *i.e.* X-rays, radium and electrical treatment, are fully described, but the details of technique are not given. The book is well produced and the illustrations are excellent. One can recommend this book with confidence.

**The Elements of Medical Treatment.** By ROBERT HUTCHISON, M.D., F.R.C.P. Second Edition. Pp. 188. Bristol: John Wright & Sons Ltd. 1932. Price 5s. net.—The second edition of Hutchison's *Elements of Medical Treatment* has been enlarged by the addition of chapters on Physiotherapy and Anthelmintics and by a new section on Sedatives. The chapters on Anæmia and on Diabetes have been considerably revised to keep abreast of progress. Dr. Hutchison might improve his first chapter on "Some General Principles" by bringing his instructions for signing prescriptions into keeping with the intricacies of the Dangerous Drugs Act: there are some occasions when the mere initials of the doctor will not suffice. He is, in our opinion, unwise to make jest at the full formula for a drug like veramon, for he has no reason to suppose that a modern medical student will not appreciate the meaning of the formula; indeed, it is a pity that the formula is not quoted in many other examples of these synthetic drugs with proprietary names. It is surely by an oversight that Mist. Sennæ Co. appears under the "Anthracene Group" of aperients only, and that no mention is made of the "Saline" it contains. Some of the modern drugs for relieving cardiac and renal dropsy, like novasurol, salyrgan and neptal, might well have been mentioned in this revision, and the omission of mercurochrome from the urinary antiseptics is most striking. In the chapter on hæmorrhage the value of intramuscular and intravenous injection of citrates is ignored. The instructions for treating diabetes lay too much stress on diet ("the main line of attack is by diet") and too little on the use of insulin in enabling the patient to take a normal diet. The author says, "Diabetic foods are less necessary now," but he continues to advocate a group of expensive "foods" which have the additional demerit of being unappetising. The dangers of an excess of fat are not referred to. But the bad old practice of fast-days is apparently not even now wholly condemned by the author. The book is not one which can be recommended as likely to guide the student and newly-qualified practitioner to the most modern advances in treatment, and may even prove misleading in the application of some old and established plans.

**Obstetrics and Gynæcology.** By JOSEPH B. DE LEE, M.D., and J. P. GREENHILL, M.D., F.A.C.S. Pp. 665. Illustrated. Chicago: The Year Book Publishers. 1931.—This is one of the Practical Medical Series for 1931, and consists of préces by the authors of all the latest work in connection with the two subjects of gynæcology and obstetrics. The work and views of American authors form at least three-quarters of the matter reviewed, and a large proportion of this is typical of the "meddlesome midwifery" of that country. Such, indeed, is the view expressed on several occasions by the authors. In spite of this drawback, there is reviewed a sufficient proportion of excellent experimental work to make the book well worth reading by those who specialize in these subjects.

**The Breast-fed Baby in General Practice.** By LESLIE GEORGE HOUSDEN, M.B. Pp. ix., 118. London: H. K. Lewis & Co. Ltd. 1932. Price 4s. 6d.—This book contains the "Studies in Breast Feeding" awarded the Sir Charles Hastings Clinical Prize, 1932, by the British Medical Association. It is a most useful and helpful book, and the subject is vital to the interests of the child, and often to the reputation of the practitioner. The case for breast feeding is presented in a simple, convincing, and stimulating fashion, and should be of great assistance in enabling the doctor to institute natural feeding in those cases where indifference or resistance is shown by the patient, nurse or relatives. Breast feeding is often abandoned for artificial methods because of the various difficulties and anxieties which may occur during the early months, and many practical points are discussed. The chapters on maternal diet and the feeding of premature infants, twins, and triplets are also of great value.

**The Infant.** By W. J. PEARSON, D.M., and A. G. WATKINS, M.D. Pp. vii., 47. London: H. K. Lewis & Co. Ltd. 1932. Price 2s. 6d. This book presents a brief epitome of the essential points to be observed in the feeding and management of infants. The food-value tables and caloric requirements will prove particularly useful, so too will the "landmarks" in child development. The book lacks an index, and we have failed to find any reference to the protein requirements of infants or the best forms of protein foods. On the whole, however, those infant welfare workers (lay and medical) for whom it is intended will find it a reliable reference hand-book, so too will any doctor who has to give advice on the management of infants.

**Injection Treatment in Medical Practice.** By DAVID LEVI, M.S. Pp. vii., 150. Illustrated. London: Cassell & Co. Ltd. 1932. Price 6s.—One might not have thought that "injection treatment" could form the sole subject of a book. But Dr. Levi has collected a surprising number of remedies which are administered by this method. His little volume fully justifies the undertaking. Piles, varicose veins, local anæsthesia in fractures, neuralgias, delivery of retained placenta and blood transfusion are some of the conditions whose treatment by means of injections are described. His technique is sound and his descriptions are clear. This is likely to prove a most valuable guide in general practice.

**The Francis Treatment of Asthma.** By A. FRANCIS, M.B. Pp. viii., 160. London: William Heinemann Ltd. 1932. Price 7s. 6d.—The Francis treatment of asthma consists in light cauterization of the nasal septum. The lightest possible touch is required, such as produces neither swelling nor irritation. By such cauterization our author can at will raise or lower the blood-pressure. Asthma is due to instability of the vasomotor system. (And since "vasomotor disturbance is the cause of most ailments," the same treatment will cure migraine, angina pectoris, mucous colitis and Raynaud's disease.) The author utters a special warning against removing nasal polypi in asthmatic subjects, above all in those persons who cannot take aspirin. More than half the book is occupied by reprints of papers previously published by the author (or submitted for publication but rejected) and by reports of 400 treated cases. Those who are interested in the subject of asthma will probably find it worth reading, and will be able to assess the value of the evidence.

**Practical Food Inspection.** Vol. I. By C. R. A. MARTIN. Pp. vii., 312. Illustrated. London: H. K. Lewis & Co. Ltd. 1932. Price 15s.—Too often books on such matters as food inspection are written by men with little practical experience of the administrative difficulties associated with the duties. If for no other reason, this book, by an inspector of many years' experience, is a valuable contribution to the literature upon the subject. The book is well arranged in chapters, and deals with the subject from physiology and anatomy to ante-mortem inspection, the actual slaughtering, meat inspection and pathological conditions, including infectious and parasitic diseases. A chapter at the end deals with the preservation of meat. There is an excellent glossary of the more uncommon terms and an index at the end of the book. The book will be most valuable to any Health Department, and students who desire a clear conception of the subject of meat inspection cannot do better than study it.

**Massage and Remedial Exercises.** By NOËL M. TIDY. Pp. xii., 429. Illustrated. Bristol: John Wright & Sons Ltd. 1932. Price 15s.—This is in many respects an admirable book, as it is arranged most methodically, is most generously and efficiently illustrated, and includes the most modern ideas and methods in a large number of branches. On the other hand, it seems likely to prove wearisome to the careful student, because it crowds an enormous material into relatively few pages by means of a small type. If the candidate for the C.S.M.M.G. diploma is really expected to be familiar with the details of pathology which are included in this book, one can only deplore the attitude of the examiners who demand knowledge of so little practical use to a masseuse. One looks in vain in this book for the illuminating comment of the experienced practitioner guiding the novice among the maze of exercises and tests, which are doubtless necessary to the examination candidate, but demand pruning and selection in real life. There is a good index.

**General Nursing.** By ALAN PERRY, F.R.C.S., and DOROTHY HARVEY. Pp. viii., 408. Illustrated. London: Edward Arnold & Co. 1932.—This small book is intended to cover the syllabus of the State examination for nurses, and it is written on the assumption that its readers are undergoing a practical course of instruction. Certainly the amount of information contained in the book is very remarkable, and it is conveyed in such a way as to be both clear and interesting. The illustrations are clear and well drawn, and we feel sure that the book will be a most welcome friend and help to the nurse in her first year of work. It will probably also serve a useful purpose in giving an idea of elementary anatomy, physiology, surgery, and medicine to school teachers.

**Materia Medica for Nurses.** Second Edition. By A. MUIR CRAWFORD, M.D. Pp. viii., 89. London: H. K. Lewis & Co. Ltd. 1933. Price 3s. 6d.—Professor Crawford has brought out a second edition of his useful book on materia medica for nurses so as to bring it into accordance with the last edition of the British Pharmacopœia. Medical students as well as nurses will find it worth their while to read this admirable and practical summary of drugs and doses. It has the great merit of mentioning only the really important preparations which are in constant use.

